# The Screening of Potassium- and Phosphate-Solubilizing Actinobacteria and the Assessment of Their Ability to Promote Wheat Growth Parameters

**DOI:** 10.3390/microorganisms9030470

**Published:** 2021-02-25

**Authors:** Kenza Boubekri, Abdoulaye Soumare, Ilham Mardad, Karim Lyamlouli, Mohamed Hafidi, Yedir Ouhdouch, Lamfeddal Kouisni

**Affiliations:** 1 Laboratory of Microbial Biotechnologies Agrosciences and Environment (BioMAgE), Faculty of Sciences Semlalia, Cadi Ayyad University, Marrakech P.O. Box 2390, Morocco; Kenza.BOUBEKRI@um6p.ma (K.B.); ouhdouch@uca.ac.ma (Y.O.); 2 AgroBioSciences Program, Mohammed VI Polytechnic University (UM6P), Benguerir 43150, Morocco; Abdoulaye.SOUMARE@um6p.ma (A.S.); ilham.mardad@um6p.ma (I.M.); karim.LYAMLOULI@um6p.ma (K.L.); Lamfeddal.KOUISNI@um6p.ma (L.K.)

**Keywords:** Actinobacteria, rock phosphate, potassium, solubilization, biofertilization, PGP traits, wheat germination

## Abstract

Soil fertility and plant nutrition require an adequate management of essential macronutrients such as potassium (K) and phosphorus (P), which are mandatory for plant development. Bioleaching of K and P bearing minerals improves their chemical weathering and increases the performance of the biofertilization strategies. In this study, in vitro and greenhouse experiments were carried out to investigate P and K solubilization traits of nine Actinobacteria (P13, P14, P15, P16, P17, P18, BC3, BC10, and BC11) under fertilization with rock phosphate (RP). K and P solubilization were evaluated on Alexandrov and NBRIP media containing mica and six RP samples, respectively. The actinobacterial strains were able to solubilize K in Alexandrov medium supplemented with RP. However, when soluble P was used instead of RP, only four strains of Actinobacteria (*Streptomyces alboviridis* P18–*Streptomyces griseorubens* BC3–*Streptomyces griseorubens* BC10 and *Nocardiopsis alba* BC11) solubilized K. The solubilization values of K ranged from 2.6 to 41.45 mg/L while those of P varied from 0.1 to 32 mg/L. Moreover, all strains were able to produce IAA, siderophore, HCN, and ammonia and significantly improved the germination rate and the vigor index of wheat. The pot experiments revealed that four strains (*Streptomyces alboviridis* P18, *Streptomyces griseorubens* BC3, *Streptomyces griseorubens* BC10, and *Nocardiopsis alba* BC11) significantly improved the growth parameters of wheat, namely root length (1.75–23.84%), root volume (41.57–71.46%), root dry weight (46.89–162.41%), shoot length (8.92–23.56%), and shoot dry weight (2.56–65.68%) compared to the uninoculated control. These findings showed that *Streptomyces griseorubens* BC10 and *Nocardiopsis alba* BC11 are promising candidates for the implementation of efficient biofertilization strategies to improve soil fertility and plant yield under rock P and rock K fertilization.

## 1. Introduction

In many developing countries, agriculture is a key socioeconomic driver and one of the most important sources of employment and income. With roughly 83 million people being added to the world’s population every year, it is estimated that the total demand for food will increase by 40% by 2030 and 70% by 2050 [1]. One of the most widely grown crops to meet this demand is wheat, which (after rice) is considered in the developing world as the second-most important food source since it provides more calories and proteins (20%) than any other crop [2]. Wheat demand is expected to increase by 60%. Therefore, achieving such a goal will require the implementation of efficient and sustainable fertilization approaches to improve bioavailability of essential nutrients such as phosphorus (P) and potassium (K). In fact, P and K positively affect plant metabolisms and resistance to biotic and abiotic stresses, while leading to a better soil fertility and crop production [3,4,5]. According to Mitra et al. [6] P availability also improves other vital plant functions such as cell division, cell enlargement, and transformation of starch and sugars. In this context, the direct application of rock phosphate (RP) and rock potassium (RK) is a promising solution to lower the cost and provide a sustainable complement to conventional fertilization practices, which are not always adapted to the specificities of the African agriculture [7]. Nonetheless, the low solubility of P and K is a major drawback to their direct application, notably in nonacidic soils [5,6]. Currently, converting the insoluble portion of P into soluble fraction is a key objective in sustainable agriculture [8]. Several techniques and strategies have been recently proposed to increase P solubilization and subsequently increase the P availability. The soil application of phosphate-solubilizing microorganism (PSM)-based inoculum is a promising approach, which takes advantage of the capacity of PSMs to assimilate phosphorus for their own requirement, while making it available for plant uptake [4,8,9,10]. Actinobacteria or Actinomycetes are considered as plant growth-promoting rhizobacteria (PGPR) and their P solubilization capacity has been well documented [11,12,13,14]. These filamentous bacteria have the ability to persist in very difficult and competitive environments by producing spores that adhere to soil particles [15,16]. Moreover, several interesting properties of these microorganisms have been evidenced such as the production of metabolites that improve plant growth and tolerance to biotic and abiotic stresses [17,18,19], which make them suitable candidates for the production of highly versatile biofertilizers [14,20,21,22]. Unfortunately, until now Actinobacteria have been scarcely investigated compared to other plant growth-promoting rhizobacteria such as Proteobacteria and Firmicutes [23,24,25]. Consequently, the current study focused on the evaluation of the ability of Actinobacteria strains isolated from contrasting environment to solubilize RK and different RPs, as well as their ability to produce PGP-related compounds such as indole acetic acid (IAA), siderophore, HCN, and ammonia. Overall, the specific objectives of this study are as follows:

(i) Assess the impact of the RP type and quality on the ability of Actinobacteria to solubilize P.
(ii) Investigate the behavior of RP-solubilizing Actinobacteria under K-bearing minerals such as mica.
(iii) Validate the impact of the RP–RK–Actinobacteria combination on plant growth under greenhouse conditions.
(iv) Suggest efficient K- and P-solubilizing Actinobacteria strains as biofertilizers for sustainable improvement of plant nutrition and soil fertility.

## 2. Materials and Methods 

### 2.1. Microbial Strains

The nine Actinobacteria strains that were used in this study belong to the microbial collection of the Biotechnology Laboratory at Faculty of Science, Cadi Ayyad University of Marrakech. They were isolated from two different sites of Morocco (desert soils).

Their taxonomic position showed that they belong to *Streptomyces* and *Nocardiopsis* genera (Table 1). The strains were labeled as follows: P13, P14, P15, P16, P17, P18, BC3, BC10, and BC11.

### 2.2. Sampling and Characterization of Rock Phosphate

RP samples were collected from six different sites in Morocco (RP1, RP2, RP3, RP4, RP5, and RP6). From each site, three samples were taken, each from 25 m × 25 m surface areas, thereby representing three replicates with approximatively 10 m between each replicate. A blend RP sample was taken from each area, consisting of 10 g core samples of 1 kg wet weight. They were then homogenized, sieved (<2 mm), and placed in a sterile polyethylene bag. The element composition was identified by using scanning electron microscopy (SEM/EDS) (Stereoscan 260, Cambridge, England).

### 2.3. Screening for Rock Phosphate Solubilization

#### 2.3.1. Qualitative Methods

The phosphate-solubilizing activities of each Actinobacteria strain were evaluated after transferring 10 μL of an overnight culture of each strain on NBRIP agar plates medium containing 10 g glucose, 0.1 g (NH_4_)_2_SO_4_, 0.2 g KCl, 0.25 g MgSO4·7H_2_O, 5 g MgCl_2_·6H_2_O, and 5 g of RP as a sole source of phosphorus [26]. The growth and diameter of the solubilization halo were determined after incubation at 28 °C for 11 days. pH variations in the media were shown using bromocresol purple as a dye marker. Phosphate solubilization index (PSI) was measured and calculated according to the following formula [27]:

(1) $$\mathrm{PSI}\;=\;\frac{Colony\;diameter+Diameter\;of\;halo\;zone}{Colony\;diameter}$$

#### 2.3.2. Ability of the Actinobacteria Strains to Dissolve Rock Phosphates in Liquid Medium

To remove the soluble fractions, RP samples were washed four times with distilled water, oven-dried at 60 °C for 2 h, and then homogenized before use. The RP solubilization ability was assessed on liquid NBRIP media containing 5 g of each RP samples as the sole source of P. Briefly, 1 mL of each actinobacterial suspension was individually inoculated in 50 mL of NBRIP broth and incubated at 28 °C, 160 rpm. After 3, 7, and 11 days of incubation, 1 mL of each culture was collected in three replicates and centrifuged at 10,000 rpm for 10 min. Non-inoculated sterilized broth was used as the control. The soluble P was determined according to Nagul et al. [28] and the pH variation was recorded at T0, T3, T7, and T11.

### 2.4. Screening for Rock Potassium Solubilization

K solubilization was evaluated on Alexandrov agar medium (containing per L: 5 g glucose, 0.5 g MgSO_4_·7H_2_O, 0.1 g CaCO_3_, 0.006 g FeCl_3_, 2 g KH_2_PO_4_, 20 g agar, and 5 g of mica as an insoluble source of potassium) [29], and using mica (2.35% CK; 2.15% NK; 45.94% OK; 1.07% NaK; 1.13% MgK; 19.45% AlK; 18.22% SiK; 0.04% PK; 0.50% SK; 7.71% KK, and 1.44 % CaK) as the sole K source. Alexandrov agar plates were inoculated with 10 µL of each Actinobacteria strain and incubated at 28 °C for 11 days. Likewise, the quantitative estimation of potassium solubilization rate was carried out on Alexandrov liquid media supplemented with mica, by adding 1 mL of a 48h culture of each strain to 100 mL of Alexandrov broth. After 11 days, K release in inoculated and non-inoculated treatments was evaluated by atomic absorption spectrometry according to Othman et al. [30].

### 2.5. Dual Solubilization of Potassium and Phosphate 

To assess the ability of Actinobacteria strains to simultaneously solubilize K and P, Alexandrov broth containing RP and mica as the sole source of P and K was used. An amount of 10 µL of each pure culture was suspended in 100 mL of broth. The cultures were incubated at 28 °C for 11 days. The available K and P in the supernatant were evaluated using the previously described protocol. 

### 2.6. In Vitro Evaluation of Other Actinobacteria PGP Traits

#### 2.6.1. Indole Acetic Acid (IAA) Production

Auxin production by different Actinobacteria strains was determined colorimetrically according to Sachdev et al. [31]. Briefly, the culture media was centrifuged (11,000 rpm, 15 min), then 1 mL of supernatant was added to 2 mL of Salkowski reagent. After 30 min of incubation, the appearance of the color pink was an indicator of a positive production of IAA. The absorbance was read at 530 nm [32]. The level of IAA production was calculated based on the calibration curve of IAA and the results were expressed as µg of IAA per mL of the extract.

#### 2.6.2. Hydrogen Cyanide Production

The ability of Actinobacteria to produce HCN was tested for each strain in overnight Luria–Bertani (LB) broth supplemented with glycine (4.4 g/L). The qualitative test was determined by putting underneath each Petri dish lids a Whatman filter paper flooded for 1 min by a solution of 0.5% picric acid in 2% sodium carbonate and incubated for 1 week at 28 °C. A change from yellow to orange/red on the Whatman filter paper indicates a positive production [33]. The results were then devised into three groups depending on their intensity of production (e.g., weak (+), moderate (++), or strong (+++)).

#### 2.6.3. Ammonia Production

An overnight culture of each Actinobacteria strain was inoculated into 1 mL of peptone water and incubated at 28 °C, 120 rpm for 11 days. Nessler’s reagent (0.5 mL) was then added in each tube and the ammonia production was detected by the development of a yellow-brown color [34].

#### 2.6.4. Siderophore Production

Siderophore production was evaluated using the Chrome Azurol S (CAS) reagent [35]. The CAS assay was performed according to a modified method of Lynne et al. [36]. A mixture of 100 mL of CAS reagent and 900 mL of sterilized Luria–Bertani (LB) agar medium was used for siderophore detection in CAS agar plates. Each Actinobacteria strain was spotted on each plate and incubated at 28 °C for 11 days. An uninoculated plate was used as control. After incubation, the formation of an orange zone around the colonies was reported as positive. 

### 2.7. Effect of Actinobacteria Strains on Wheat Germination: In-Vitro Tests

A pure bacterial colony from each Actinobacteria strain was suspended into 50 mL of LB medium, in order to prepare the inoculums. The Actinobacteria strains were cultivated for 10 days in LB broth and incubated at 28 ± 30 °C at 160 rpm. For the seed preparation, durum wheat seeds (*cv. Vitron*) were surface sterilized using 98% ethanol (30 s) and 2% sodium hypochlorite (2 min) and successively washed with distilled water. Then, 20 seeds of wheat were placed in sterile Petri dishes (100 mm × 15 mm) containing agar media (0.8% of agar) and inoculated with 48 h of each actinobacterial culture (1 mL) containing 10^8^ UFC/mL [37]. The plates were incubated at 28 °C for 11 days. The experiment was arranged according to a complete randomized block design with three replicates and four treatments: (1) negative control; (2) control with RP; (3) control with the strain; and (4) strain + RP. The germination parameters such as percent of germinated seeds, radicle, and plumule length were measured after 11 days and the vigor index was then calculated.

### 2.8. Greenhouse Experiment

Based on the in vitro assays results, the top four performing Actinobacteria (*Streptomyces alboviridis* P18, *Streptomyces griseorubens* BC3, *Streptomyces griseorubens* BC10, and *Nocardiopsis alba* BC11) were selected to evaluate their capacity to release, under greenhouse conditions, P and K from RP and mica, respectively. The experiment was conducted at the experimental farm of the Mohamed VI Polytechnic University in Benguerir, Morocco, from September to November 2020. Wheat (*Triticum aestivum* variety *Vitron)* was grown in PVC pots (150 mm height, 80 mm diameter) containing 1.5 kg of sterilized mixture of sand (with low P) and perlite (3:1 *w*/*w*). Before sowing, seeds were first surface sterilized with 2% (*v*/*v*) sodium hypochlorite for 1 min, 95% (*v*/*v*) ethanol for 30 s, and then washed with sterile distilled water for 1 min (three times) [37]. Pots (four seeds per pot) were arranged according to a completely randomized block design with five replicates and six treatments: (1) (C−) negative control (without bacterial inoculation, Mica, nor RP fertilization); (2) C+ (TSP) positive control containing triple superphosphate (containing 46 % soluble P_2_O_5_); (3) treatment N3 fertilized with mica; (4) treatment N4 containing RP (32.5% total P_2_O_5_); (5) treatments N5 containing Mica + RP; and (6) treatment N6 with four co-inoculations containing RP + mica each with the strains (*Streptomyces alboviridis* P18, *Streptomyces griseorubens* BC3, *Streptomyces griseorubens* BC10, and *Nocardiopsis alba* BC11). Microbial inoculation was performed by adding 2 mL of each Actinobacteria suspension (OD = 1) in the vicinity of the root zone. Plants were grown for 60 days and watered two times per week with half-modified Hoagland solution [38] without any sources of P and K.

### 2.9. Plant Analysis 

At the end of the experiment, the plants were carefully taken out of the pots and washed with distilled water to remove adhering particles. Then, root traits were determined using WinRHIZO image analyzing system (Regent Instructions, Quebec, Canada). Shoot and root dry weight was determined after overdrying at 68 °C for three days. Thereafter, the estimation of the plant response to inoculation with the Actinobacteria strains in terms of biomass enhancement was calculated using the following formula:

(2) $$\mathrm{GE}\;(\%)\;=\;\frac{\left(\mathrm{Shoot}\;\mathrm{dry}\;\mathrm{weight}\;\mathrm{of}\;\mathrm{treated}\;\mathrm{plants}\;-\;\mathrm{shoot}\;\mathrm{dry}\;\mathrm{weight}\;\mathrm{of}\;\mathrm{control}\;\mathrm{plants}\right)}{\mathrm{Shoot}\;\mathrm{dry}\;\mathrm{weight}\;\mathrm{of}\;\mathrm{control}\;\mathrm{plants}}\;\times \;100$$

where GE denotes growth enhancement.

### 2.10. Statistical Analysis

All the results were statistically analyzed using IBM SPSS Statistics 20 Software. A comparison between treatments was performed using one-way analysis of variance (ANOVA) with least significant difference (LSD). Tukey’s comparison test was performed at *p* = 0.05 in case of significant impact by factor. The heatmap and PCA were built using an in-house script using the Scikit-Learn Python library. 

## 3. Results

### 3.1. Rock Phosphate Composition and Solubilization Index 

Based on colony diameter and halo zone, solubilization index (SI) was calculated for each strain. The results are presented in Table 2. Among all tested Actinobacteria, the strain *Streptomyces anulatus* P16 shows the highest solubilization index (SI = 3.17) on NBRIP solid medium. 

### 3.2. Rock Phosphate Solubilization by the Actinobacteria Strains 

**Figure 1:**
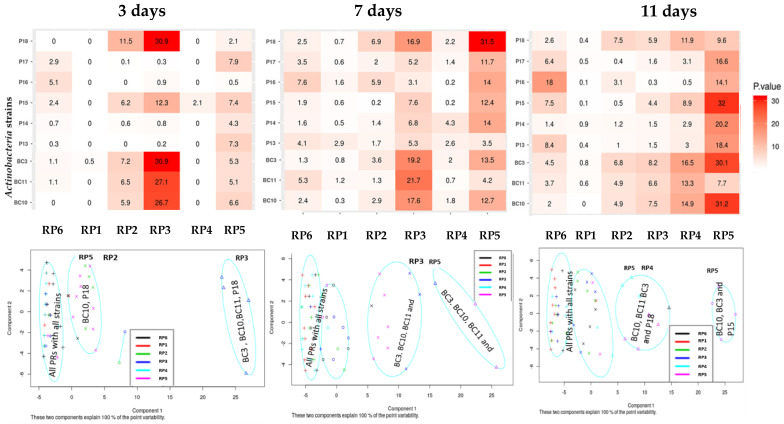
Heatmap and PCA of Actinobacteria strains solubilization as a function of different grade of RP after 3, 7, and 11 days of incubation (T1, T2, and T3). The values represent mean (*n* = 3) corresponding to the RP solubilization activity. As shown in the color scale, red indicates a high RP solubilization. The signs x, o, + and Δ in the ellipses correspond to solubilization rates.

The nine studied Actinobacteria strains endowed different abilities to release soluble phosphate from the six different RP samples. The RP solubilization potential of the strains varied according to the incubation time and the RP type. Overall, the major difference between these types of RPs was in P_2_O_5_ and SiO_2_ content. All strains reached their maximum solubilizing capacity after 11 days of incubation regardless of the RP type (Figure 1). The solubilization of the six RP samples by the Actinobacteria strains ranged from 0.1 to 32 mg/L. The most performing strains were respectively *Streptomyces alboviridis* **P18**, *Streptomyces griseorubens* **BC3**, *Streptomyces griseorubens* **BC10**, and *Nocardiopsis alba* **BC11**. The Actinobacteria strains showed a broad RP solubilization spectrum. In general, all strains were able to dissolve at least one RP type. The phosphate-solubilizing capacity was dependent on RP P_2_O_5_ content and chemical composition. Furthermore, the heatmap (Figure 1) showed the highest solubilization activity of *Streptomyces griseorubens* BC3, *Nocardiopsis alba* BC11, *Streptomyces griseorubens* BC10, and *Streptomyces alboviridis* P18 on RP3 and RP5 after 3 and 7 days of incubation. After 11 days of incubation, the strains *Streptomyces alboviridis* P18, *Streptomyces griseorubens* BC3, *Streptomyces griseorubens* BC10, *Nocardiopsis alba* BC11, and *Streptomyces microflavus* P15 showed a high solubilization efficiency for RP5 followed by RP4. The other strains exhibited low rates of solubilization regardless of the RP type and the incubation time. 

### 3.3. Variations of pH during the Solubilization of Various RP Samples by the Actinobacteria Strains

**Figure 2:**
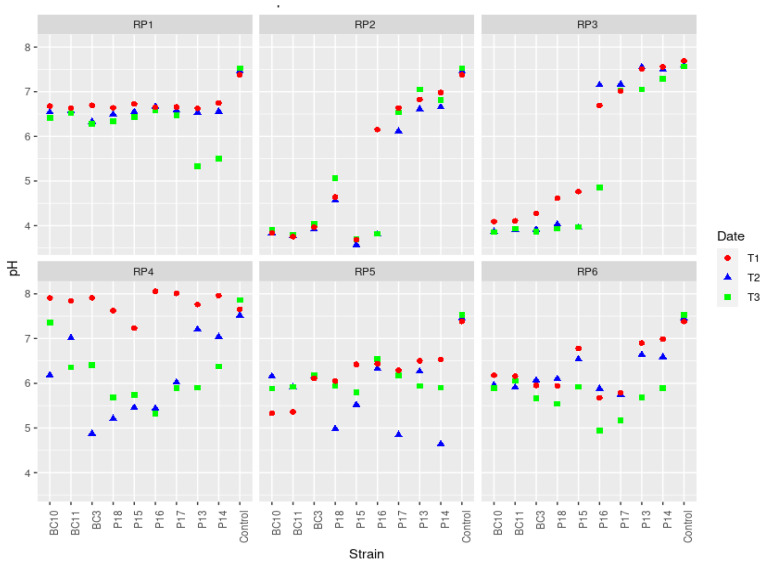
Changes in pH during the solubilization of different types of RPs by Actinobacteria strains. T1, T2, and T3 correspond to the measurements carried out after 3, 7, and 11 days of incubation, respectively.

The pH variation was dependent upon both the RP types and the strain used (Figure 2). For the Actinobacteria strains *Streptomyces griseorubens* BC3, *Streptomyces griseorubens* BC10, *Nocardiopsis alba* BC11, *Streptomyces alboviridis* P18, and *Streptomyces microflavus* P15, the pH was slightly acidic (~6) during the solubilization of RP1, RP6, RP4, and RP5, but it was very acidic (~4) for RP2 and RP3. On the other hand, Actinobacteria strains *Streptomyces fulvissimus* P13, *Streptomyces youssoufiensis* P14, *Streptomyces pratensis* P17, and controls showed a pH ranging from 6 to 7.5 (Figure 2) when they were tested on PR2, RP3, and RP4. For the RP5 rock, all the strains acidified the culture medium. The pH of the control media remained globally neutral (~7.5).

### 3.4. Solubilization of Potassium by the Actinobacteria Strains

**Figure 3:**
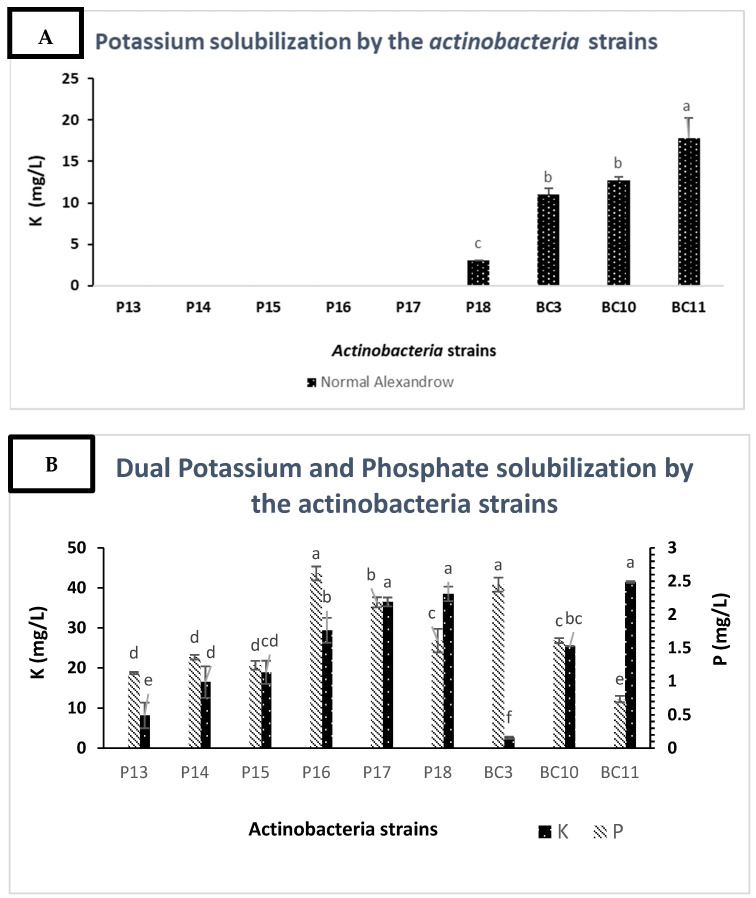
(**A**) Potassium solubilization by the selected Actinobacteria strains after 7 days of incubation. (**B**) Potassium solubilization by the Actinobacteria strains under phosphorus deficiency. The bars represent the mean values ± SD of three replicates. For each mineral, the mean values followed by different letters are significantly different according to Tukey (*p* = 0.05).

Among all investigated Actinobacteria strains, only *Streptomyces alboviridis* **P18,** *Streptomyces griseorubens* **BC3,** *Streptomyces griseorubens* **BC10**, and *Nocardiopsis alba* **BC11** were able to solubilize mica as a sole source of potassium (Figure 3A). The values ranged from 3 to 17.8 mg/L of potassium. However, on the modified Alexandrov medium, with rock phosphate and mica as a source of insoluble phosphorus and potassium, all strains were able to solubilize the insoluble form of potassium. The solubilization rate ranged from 2.6 to 41.45 mg/L of potassium (Figure 3B). In both conditions, the *Nocardiopsis alba* strain BC11 was found to be the best performing Actinobacteria in terms of potassium solubilization (41.45 mg/L). 

### 3.5. PGP Traits of Selected Actinobacteria Strains

#### 3.5.1. IAA Production 

The production of IAA by the Actinobacteria strains was estimated because of their important roles in plant growth promotion and development. The tested strains were able to produce IAA at a concentration of 2.75 to 128.44 mg/L after 11 days of incubation. The highest IAA production was achieved by *Streptomyces griseorubens* BC10 (128.44 mg/L) followed by *Nocardiopsis alba* BC11 (82.33 mg/L), while the lowest IAA production was obtained by *Streptomyces fulvissimus* P13 (1.73 mg/L) (Table 3).

#### 3.5.2. HCN, Ammonia, and Siderophores Production

**Figure 4:**
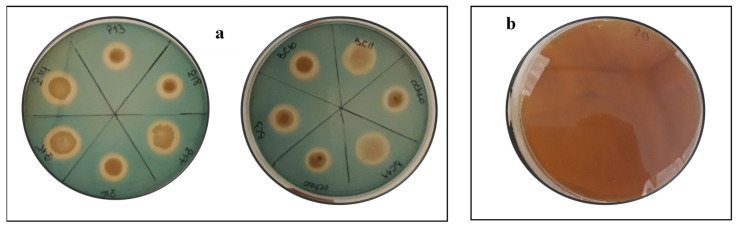
Siderophore production on Chrome Azurol S (**a**) and HCN production (**b**). Note: Positive results are indicated by the formation of a halo zone around the colonies for siderophore production and a change of brown color for HCN production.

Picric acid assay was used to determine the ability of the Actinobacteria strains to produce HCN. All strains were able to produce HCN at different levels (Table 3). Eleven percent of the tested strains showed strong production of HCN (+ + +), while 66% showed moderate production (+ +) and 33% showed weak production (+) (Table 3). In addition, the ability to produce ammonia by selected Actinobacteria was contrasting (Table 3). Ultimately, all the evaluated strains demonstrated the ability to produce siderophore as evidenced by the formation of a distinct halo zone around the colonies on CAS agar media (Figure 4). 

### 3.6. Germination and Vigor Index Improvement in Wheat Seedlings

**Figure 5:**
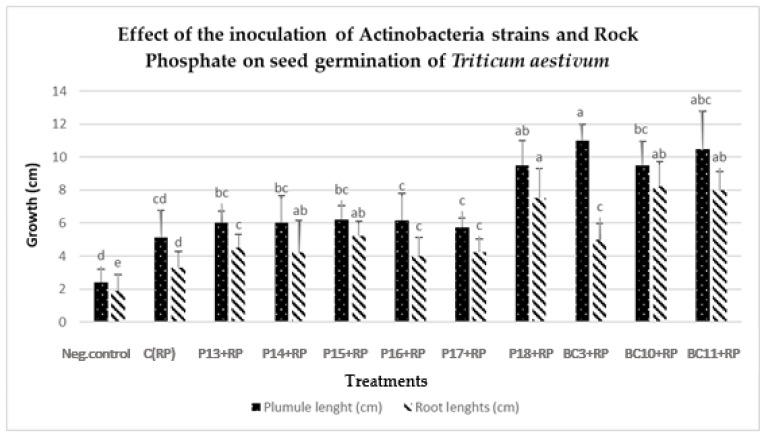
Effect of the combination of rock phosphate and Actinobacteria strains on the plumule and root of *Triticum aestivum* seeds. The bars represent the mean values ± SD of five replicates. The different letters indicate the existence of significant differences according to Tukey (*p* = 0.05).

**Figure 6:**
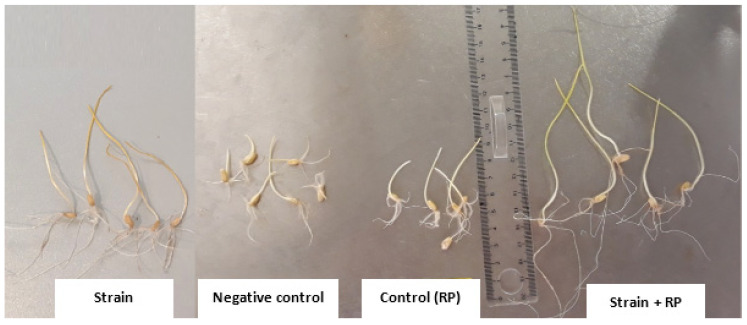
Effect of the combination of RP with Actinobacteria strains on wheat seed germination.

The effect of the Actinobacteria strains on wheat germination parameters was carried out for RP5 since it was the best-performing RP regarding P solubilization under in-vitro microbial experiments. All treatments showed a significant positive effect on the germination rate and vigor index compared to the negative control (Table 4). The inoculated wheat seedlings (solely or in combination with RP) revealed a significant increase of seed germination at *p* < 0.05 compared to non-inoculated control, which had the lowest rate of germination (80%) (Table 4 and Table 5, Figure 5 and Figure 6). 

For both germination rate and vigor index, the maximum increase was obtained by the strains *Nocardiopsis alba* **BC11**, *Streptomyces griseorubens* **BC10**, and *Streptomyces alboviridis* **P18** at 18.5%, 17.75%, and 17%, respectively, compared to the treatments with strains only (Table 5). The growth of the wheat plantlets was much enhanced by the addition of the RP. In fact, the combination of Actinobacteria strains and rock phosphate increased the length of hypocotyl of wheat seed by 86.44% and 101.21% of the length roots compared to the treatments with strains only (Table 4 and Table 5, Figure 5 and Figure 6).

### 3.7. Greenhouse Trials

**Figure 7:**
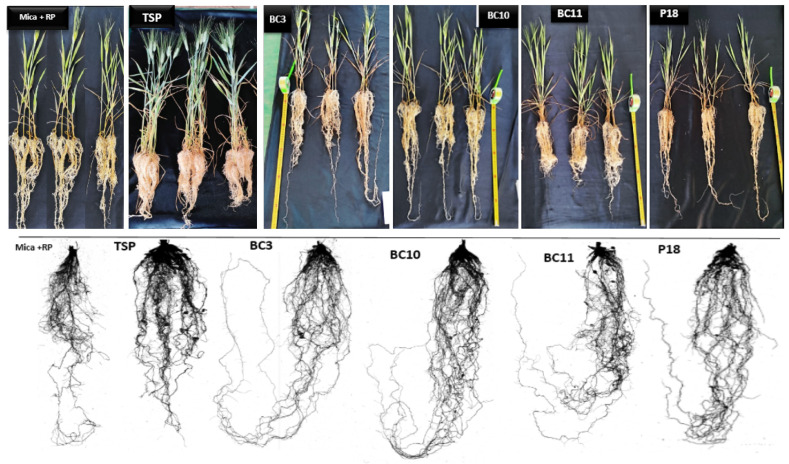
The effect of Actinobacteria inoculation on root growth architecture of the wheat plant.

Under greenhouse conditions, the positive control treated with TSP had significantly higher shoot length and shoot dry weight than all the other treatments. However, the selected Actinobacteria strains significantly enhanced all the plant growth parameters—plant height (8.92–23.56%), root length (1.75–23.84%), shoot dry weight (2.56–65.68%), and root volume (41.57–71.46%)—over the control (mica + RP). The wheat plants inoculated with *Streptomyces griseorubens* BC10 showed the maximum shoot lengths (40.5 cm/plant) and root dry weight (0.761 g/plant) compared to the uninoculated control. In addition, all the selected strains significantly increased (*p* < 0.05) root length ranging from 57.31 to 69.75 cm/plant compared to the uninoculated control (56.32 cm/plant). The *Nocardiopsis alba* strain BC11 recorded the highest root length (69.75 cm/plant). The shoot and root dry weights, as a direct measure of plant growth parameters, were clearly increased by different Actinobacteria inoculations. In general, among the tested strains, *Streptomyces griseorubens* BC10 and *Nocardiopsis alba* BC11 showed a coarse root architecture and the highest agronomic performance of wheat plant (Table 6 and Figure 7).

## 4. Discussion

Poor soil fertility is one of the most important constraint-limiting crop yields in developing countries [39]. Fortunately, it can be significantly improved by adopting sustainable approaches such as using beneficial rhizobacteria (e.g., PSMs, nitrogen-fixing bacteria) as biofertilizers. Among PSMs, Actinobacteria are known to be eco-friendly and efficient plant growth promoters [24]. Therefore, the main objective of this study was to focus on Actinobacteria strains’ abilities to dissolve mica and six different RPs. The phosphate solubilization index (SI) was assessed on NBRIP agar containing RP as the sole source of phosphorus. Most of the strains showed a positive SI, except *Streptomyces alboviridis* P18 (Table 2). The highest SI (3.17) was recorded for *Streptomyces anulatus* strain P16, which also showed a high RP solubilization rate in a liquid medium. Interestingly, *Streptomyces alboviridis* strain P18 showed a strong solubilization capacity in liquid media (Figure 1) despite the absence of a solubilization halo in solid media, which indicates the occurrence of solubilization mechanisms other than acidification [40]. In fact, similar findings were reported by Djebaili et al. [41] regarding P-solubilizing Actinobacteria, as well as by Nautiyal [26], who isolated two efficient P-solubilizing *Pseudomonas*, which could not form the typical halo zone on agar plates. These results suggest that the SI derived from the solubilization halo should not be the sole criterion to take into consideration for screening efficient PSMs [38,39].

In liquid media, the relative efficiency of the nine Actinobacteria strains in dissolving the six RPs was related to the type of RP and the bacterial species (Figure 1). The solubilization rate of RP was directly correlated to RP P_2_O_5_ content. The capacities of the Actinobacteria strains to solubilize the RPs ranged from 0.1 to 32 mg/L (Figure 1). These results are in agreement with the findings of Hamdali et al. [42], who reported a high amount of phosphate-solubilizing activity by *Streptomyces griseus* and *Streptomyces cavourensis* with 29.67 and 21.43 mg/L, respectively. Similar results were also obtained by Nafis et al. [43], who reported that two *Streptomyces* isolated from the desert and mountain soils are the most efficient phosphate solubilizers (solubilization rate of 12.39 and 8.56 mg/L). Several mechanisms can be involved in microbial P solubilization, with the most common one being via media acidification, as shown by our experiment (Figure 2). The acidification is usually attributed to the production of several organic acids from the fermentation of organic compounds such as citric, gluconic, lactic, malic, and oxalic acid [44,45,46,47]. In fact, the decrease in pH enhances RP dissolution by removing Ca from rock phosphate, thus releasing P into the solution [48]. Several literature reports suggest that the solubilization of mineral phosphate by microorganisms might also be due to the production of chelating substances (e.g., siderophores) that bind with metal cations (aluminum, iron, and calcium), thus preventing phosphorus complexation [49,50]. Interestingly, all tested Actinobacteria strains were able to produce siderophores (Table 3, Figure 4), which may suggest that a dual solubilization process may be involved, implying both media acidification and siderophore secretion. 

K solubilization assays revealed that among the selected strains, only *Streptomyces alboviridis* P18, *Streptomyces griseorubens* BC3, *Streptomyces griseorubens* BC10, and *Nocardiopsis alba* BC11 were able to solubilize mica at 3, 11, 12.75, and 17.8 mg/L, respectively (Figure 3A). Microbial K solubilization has been scarcely investigated, and in our knowledge, only *Arthrobacter* sp. 42, *Arthrobacter* sp. 4, and *Microbacterium* FS-01 were reported to be K solubilizers [47]. In general, only 5% of potassium-solubilizing bacteria are Actinobacteria [51,52]. Similar to P solubilization mechanisms, K solubilization is also attributed to either media acidification or the chelation of cations that usually bind to K [51]. However, in this study, K solubilization under phosphorus deficiency showed some relevant results since all the Actinobacteria strains were able to solubilize the potassium source (Figure 3B). These results showed that the deficiency in P stimulates the solubilization of K. For instance, the K solubilization value of *Nocardiopsis alba* BC11 was 41.5 mg/L under P deficiency and 17.8 mg/L under P (KH_2_PO_4_) sufficiency. Such difference could be explained by the fact that molecules involved in P solubilization may also solubilize the insoluble source of K (mica) and, therefore, trigger more efficient K solubilization. Similar results were found by Abou-el-Seoud and Abdel-Megeed [53], who reported that co-inoculation of P- and K-dissolving bacteria under RP fertilization increased P and K availability and uptake. Besides their capacity to solubilize RP and K, the Actinobacteria strains were screened for plant growth promoting (PGP) factors, which are considered an effective tool in the investigation of microorganisms that can be used as biofertilizers. Such assays are of high importance as they enable the selection of elite strains that have the best agronomic potential [54]. All the tested strains showed notable PGP activities, such IAA, HCN, and ammonia production. The production of phytohormones such as auxin (IAA) plays an important role in stimulating the development of the rooting, and they act as signaling molecules involved in the production of secondary metabolites and Actinobacteria sporulation [55,56,57]. In the present investigation, all the Actinobacteria strains were able to produce IAA at concentrations varying between 2.75 and 128.44 mg/L (Table 3). After 11 days of incubation, *Streptomyces griseorubens* BC10 was found to be the best strain for IAA production (128.44 mg/L). These results exceeded the ones previously reported by Nafis et al. [43] (75.54 mg/L obtained by *Streptomyces* sp. MNC-1 isolated from the Merzouga Moroccan desert). The lowest IAA production was observed for *Streptomyces anulatus* strain P13 with 2.75 mg/L (Table 3). These results were in agreement with those reported by Doumbou et al. [58]. On the other hand, all the Actinobacteria strains produced ammonia and hydrogen cyanide at different levels (Table 3). These two compounds play a crucial role in the suppression of plant diseases. HCN is a volatile compound with antifungal properties, while ammonia has been reported to have a direct role in alleviating biotic stress [59,60]. 

Most of the evaluated strains had a positive effect on the germination and growth traits of wheat seedlings (Table 4, Figure 5 and Figure 6), which is in agreement with the work of Sharma et al. [60], who demonstrated that the use of PSB inoculants (*P. fluorescens* and *B. megaterium*) improves the radicle and plumule lengths by 59.7% and 56.4%, respectively, compared to non-inoculated treatments. Under greenhouse conditions, the selected Actinobacteria strains (*Streptomyces alboviridis* P18, *Streptomyces griseorubens* BC3, *Streptomyces griseorubens* BC10, and *Nocardiopsis alba* BC11) significantly enhanced several wheat growth parameters, including root length (1.75–23.84%), shoot length (8.92–23.56%), root volume (41.57–71.46%), root dry weight (46.89–162.41%), and shoot dry weight (2.56–65.68%), over the uninoculated control (Table 6, Figure 7)**.** The maximum root length was recorded with the treatments inoculated with *Streptomyces griseorubens* BC10 and *Nocardiopsis alba* BC11 (19.35 and 23.84%, respectively). Those results support our in vitro evaluation of the tested Actinobacteria strains in which a high amount of IAA was positively correlated with the improvement of plant growth parameters. In fact, these phytohormones are known to stimulate wheat germination, initiate root formation, and accelerate plant growth by enhancing root length and growth, thus enabling the plant to have greater access to soil nutrients and water [61]. Consequently, the enhancement in root length might be due to the combined effect of the higher amount of produced IAA (128.44 mg/L) by *Streptomyces griseorubens* BC10 and the mobilization of P and K from RP and mica, respectively. Interestingly, these elite strains (*Streptomyces griseorubens* BC10 and *Nocardiopsis alba* BC11) were equally efficient in improving both wheat and maize plant growth development [62]. Our findings are also in concordance with the investigation of Sreevidya et al. [63], who reported an increase in chickpea root length (17%) and shoot length (3%) following *Streptomyces* inoculation. In fact, with their abilities to produce various PGP-related molecules, *Streptomyces* are well documented in the literature [44,64]. As a hypothesis, the enhancement of the morphological parameters of wheat seeds by *Streptomyces griseorubens* BC10 and *Nocardiopsis alba* BC11 is plausibly due to their PGP traits, including IAA and siderophore production [65].

## 5. Conclusions

The evidence obtained through this study indicates that among the nine strains of Actinobacteria, *Streptomyces alboviridis* P18, *Streptomyces griseorubens* BC3, *Streptomyces griseorubens* BC10, and *Nocardiopsis alba* BC11 exhibit a significant capacity to solubilize mica and RPs under the in vitro condition, whereas under greenhouse conditions, two of these four strains, *Streptomyces griseorubens* BC10 and *Nocardiopsis alba* BC11, showed a coarse root architecture and the highest performance of wheat shoot and root growth. The ability of these strains to solubilize mica and RPs and to promote wheat growth is probably related to the organic acids, IAA, siderophores, and ammonia released by these strains. 

## Figures and Tables

**Table 1:** The strains’ molecular phylogenetic analysis based on 16S–rRNA.

Strains Code	% Sequence Identities	Actinobacteria Strains	Accession Number
**P13**	99%	*Streptomyces fulvissimus*	*MT845224*
**P14**	99%	*Streptomyces youssoufiensis*	*MT845225*
**P15**	99%	*Streptomyces microflavus*	*MT845226*
**P16**	99%	*Streptomyces anulatus*	*MT845227*
**P17**	99%	*Streptomyces pratensis*	*MT845228*
**P18**	99%	*Streptomyces alboviridis*	*MT845229*
**BC3**	100%	*Streptomyces griseorubens*	*MT845230*
**BC10**	99%	*Streptomyces griseorubens*	*MT845231*
**BC11**	100%	*Nocardiopsis alba*	*MT845232*

**Table 2:** Solubilization index (SI) of the Actinobacteria strains on solid medium.

Strain Code	Diameter of Halo Zone (mm)	Diameter of Colony (mm)	Solubilization Index
**P13**	1.6	1.4	**2.14**
**P14**	3	1.75	**2.71**
**P15**	2.1	1.5	**2.4**
**P16**	2.5	1.15	**3.17**
**P17**	5.6	3	**2.86**
**P18**	–	–	**–**
**BC3**	2.9	1.6	**2.81**
**BC10**	3.3	1.6	**3.06**
**BC11**	2.8	2.15	**2.3**

**Table 3:** AIA, siderophore, HCN, and ammonia production by the selected Actinobacteria strains. The values represent the mean values ± SD of three replicates. The different letters (a, b, c, d, e, f, g and h) indicate the existence of significant differences according to Tukey (*p* = 0.05).

Strains	AIA Production			Siderophore Production (cm)	HCN Production	Ammonia Production
	3 Days	7 Days	11 Days			
**P13**	−1.11 ± 0.40 d	−0.53 ± 0.23 f	1.73 ± 0.18 h	1.461 ± 0.00	**+ + +**	**+**
**P14**	51.45 ± 2.76 b	88.74 ± 1.17 b	57.92 ± 1.75 d	1.454 ± 0.21	**+**	**+**
**P15**	−1.5 ± 1.07 d	7.49 ± 2.24 de	16.77 ± 0.82 e	1.393 ± 0.07	**+ +**	**+ +**
**P16**	−3.64 ± 0.22 d	41.74 ± 1.77 c	57.73 ± 0.89 d	1.555 ± 0.00	**+ +**	**+**
**P17**	−2.28 ± 0.28 d	9.57 ± 0.46 d	10.61 ± 0.14 f	1.333 ± 0.21	**+ +**	**+**
**P18**	82.92 ± 1.5 a	44.31 ± 1.49 c	66 ± 0.38 c	1.477 ± 0.28	**+ +**	**+ +**
**BC3**	10.02 ± 2.34 c	2.68 ±0.5 e	3.19 ± 0.09 g	1.736 ± 0.00	**+**	**+**
**BC10**	6.30 ± 2.47 c	128.44 ± 4.08 a	72.98 ± 0.27 b	1.348 ± 0.00	**+ +**	**+ +**
**BC11**	7.57 ± 2.48 c	10.65 ± 1.41 d	82.33 ± 0.84 a	1.552 ± 0.00	**+**	**+**

The symbol + represents the positive reaction/presence. T1, T2, and T3 correspond to the measurements carried out after 3, 7, and 11 days of incubation, respectively.

**Table 4:** Inoculation effect of Actinobacteria strains on shoot and root lengths, germination rate, and vigor index. The different letters indicate the existence of significant differences according to Tukey (*p* = 0.05).

Treatments	Shoot Lengths	Root Lengths	Germination Rate *	Vigor Index
**Negative control**	2.4 ± 0.82 c	1.9 ± 0.96 d	80	
**Control (RP)**	5.14 ± 1.61 ab	3.3 ± 0.97 bcd	85	9.92%
**P13**	5.6 ± 0.54 ab	2.98 ± 0.94 cd	90	9.53%
**P14**	5.2 ± 1.75 ab	3.1 ± 0.54 bcd	85	9.76%
**P15**	4.34 ± 0.76 bc	3.4 ± 0.89 bcd	85	9.10%
**P16**	3.9 ± 0.89 bc	2.1 ± 0.41 d	90	6.66%
**P17**	5.6 ± 0.54 ab	3.7 ± 0.83 bcd	85	10.94%
**P18**	7.2 ± 0.83 a	6.5 ± 0.70 a	100	13.7%
**BC3**	5.9 ± 1.34 ab	2.6 ± 0.22 cd	100	8.5%
**BC10**	5.5 ± 0.7 ab	4.1 ± 1.19 cd	100	9.6%
**BC11**	6.1 ± 0.74 ab	4.7 ± 1.35 ab	100	10.8%

* The germination rate was measured way before the 11 days.

**Table 5:** Effect of RP phosphate solubilizing Actinobacteria co-inoculation on wheat growth.

Treatments	Effect of RP and Actinobacteria Inoculation		
	Plumule Length Increase	Root Length Increase	Vigor Index Increase
**Negative control**			
**Control (RP)**			
**P13 + RP**	+7.14%	+51%	11.66% (+2.13)
**P14 + RP**	+15.38%	+37.09%	12.05% (+2.29)
**P15 + RP**	+42.85%	+52.94%	16.17% (+ 7.07)
**P16 + RP**	+34.61%	+90.47%	9.44% (+ 2.78)
**P17 + RP**	+20.53%	+14.86%	12.64% (+1.7)
**P18 + RP**	+31.94%	+15.38%	17% (+3.3)
**BC3 + RP**	+86.44%	+92.3%	16% (+7.5)
**BC10 + RP**	+72.72%	+101.21%	17.75% (+8.15)
**BC11 + RP**	+66.66%	+70.21%	18.5% (+7.7)

**Table 6:** Effect of Actinobacteria inoculation on biomass yield and root traits of wheat.

Treatments	Shoot Length (cm)	Root Length (cm)	Shoot Dry Weight (g/plant)	Root Dry Weight (g/plant)	Root Volume (cm^3^)
**C−**	23.85 ± 0.96 d	46.15 ± 2.57 c	0.2355 ± 0.03 d	0.273 ± 0.042 c	1.12 ± 0.12 e
**C+ (TSP)**	60 ± 2.96 a	48.12 ± 6.12 bc	3.22 ± 0.35 a	1.154 ± 0.315 a	2.93 ± 0.420 ab
**Mica**	55.95 ± 1.95 a	49.56 ± 6.24 bc	2.51 ± 0.381 b	1.717 ± 0.407 a	3.131 ± 0.06 a
**C (Mica + RP)**	32.775 ± 1.77 c	56.32 ±7.41 ab	0.507 ± 0.085 cd	0.29 ± 0.45 bc	1.335 ± 0.255 cde
**RP**	32.75 ± 3.15 c	50.59 ± 7.70 bc	0.399 ± 0.08 cd	0.282 ± 0.064 bc	1.33 ± 0.18 de
**P18**	36.55 ± 2.60 bc	57.31 ± 5.06 ab	0.520 ± 0.03 cd	0.426 ± 0.066 bc	1.937 ± 0.2 bcde
**BC3**	35.7 ± 2.74 bc	68 ± 8.79 a	0.574 ± 0.083 cd	0.45 ± 0.046 bc	2.289 ± 0.68 abcd
**BC10**	40.5 ± 6.087 b	67.22 ± 9.15 a	0.82 ± 0.12 c	0.761 ± 0.147 b	2.192 ± 0.417 abc
**BC11**	39.45 ± 3.09 b	69.75 ± 1.68 a	0.84 ± 0.06 c	0.528 ± 0.049 bc	1.89 ± 0.302 cde

Data are mean values ± SD of five replicates. The different letters in the same column indicate the existence of significant differences according to Tukey (*p* = 0.05).
